# Round the Hospitals

**Published:** 1919-01-11

**Authors:** 


### ROUND THE HOSPITALS.

It is welcome news that the King has appointed Miss Sidney Jane Browne, R.R.C., Matron in Chief of the Territorial Nursing Service, to be Dame Grand Cross of the Most Excellent Order of the British Empire, for valuable services rendered in connection with the war. The past week has seen a large number of awards of the Royal Red Cross, Bar and First and Second Class, to members of the various Nursing Services, including those of the Oversea Forces, for valuable services with the armies in France and Flanders, and with the British Forces in Italy, Egypt, and Salonika. We regret we have not space to publish all the names, which will be found in the *Times* of January 2 and 4 respectively. The honours for service in France and Flanders include the award of a Bar to the Royal Red Cross to Miss M. M. Blakely, R.R.C., acting principal matron; Miss H. Hartigan, R.R.C., acting principal matron; Miss G. M. Smith, R.R.C., acting matron; Miss C. G. Stronach, acting principal matron; Miss M. M. Tunley, R.R.C., acting principal matron, all of Queen Alexandra's Imperial Military Nursing Service; thirty-nine matrons, acting matrons, sisters-in-charge, and nursing sisters are awarded the Royal Red Cross, First Class, and some 140 the Royal Red Cross, Second Class, which honour has also been conferred on twenty-seven members of Voluntary Aid Detachments. Three members of the Nursing Service of the Royal Air Force have also been awarded the Royal Red Cross (one First Class and two Second Class) in recognition of distinguished service.

Three important resolutions were passed at the last meeting of the Nursing Committee of the Royal Southern Hospital, Liverpool. The first related to the acceptance of a limited number of V.A.D. nurses with two years' consecutive training in a military hospital, and with a satisfactory recommendation from their matron, for three years'

**ROUND THE HOSPITALS**—(*continued*).

training in the hospital instead of four. There is nothing new in this concession; it has been already made by many first-class training schools in London and the provinces. But the authorities at the Royal Southern are making their concession a really attractive one by adding that these military probationers shall have the privilege of entering as first-year probationers, with the option of joining the private nursing staff later on. What deters V.A.D. members who have already for two years at least done responsible work in the wards from taking training in civil hospitals is the dread of being received and treated as though they knew absolutely nothing of the duties they have long been successfully performing under skilled supervision. Supplementary instruction they will, of course, need in such parts of the first-year course as do not enter into the practice of military hospitals. But this is a question of organisation, and we are glad to have a clear pronouncement as to status from the Royal Southern Hospital.

Considerable satisfaction, we imagine, will be felt by nurses at the Royal Southern Hospital, Liverpool, at another resolution of the Nursing Committee. For the future it has been decided that instead of granting a gold medal to the nurse who is top for the year in her junior and senior examinations and has obtained not less than 80 per cent. of marks, full fees shall be paid for her maternity training. At the same time the private nurses' salaries have been raised by £5 in each category so that their salaries commence in the first year at £35 with £5 bonus; second and third year £40 with £5 bonus; fourth year £45 with £5 bonus. In addition they are now to receive the whole of their indoor and outdoor uniform, which at present prices represents a considerable yearly accretion of income.

Officers and nursing sisters who retire from the Army on account of tuberculosis, contracted or aggravated in the course of their military service, will now be eligible for treatment, whether they are insured or not, under the National Insurance Act. This is the gist of a new Army instruction. The Ministry of Pensions will arrange for their admission to sanatoria, and pay for the treatment, deducting a certain proportion from the allowance payable to officer or nurse. The great expense of suitable sanatorium treatment forces many nurses to ignore early symptoms of the disease, and thus run disastrous risks. It is earnestly to be hoped that the accommodation alluded to in the above Army Order is of a sort acceptable to nurses. We are strongly inclined to believe that if inquiry were made, and the approximate number of tuberculous nurses could be ascertained, the desirability of establishing separate sanatoria for them would be clearly demonstrated. A suitable sanatorium for early cases would be one in which outdoor trades such as market-gardening and poultry farming could be taught. One of the most important steps to take in regard to nurses threatened with tuberculosis is to get them out of their profession, into a calling suited to their physique.

At the Marylebone Infirmary, North Kensington, the nursing staff has been reduced owing to illness and other causes to such an alarming extent that those who remain on duty are exposed to serious danger of a breakdown. The building is over-crowded, and contains already between thirty and forty more patients than it was intended to accommodate. At a time when women are being demobilised in thousands it is strange that the Marylebone Guardians have not been able to attract sufficient labour to their hospital. Like other infirmaries, it has been long working on too low a staff, and the effect of this system when any emergency occurs is apt to be catastrophic.

We are glad to note that the Committee of Management at the Great Northern Central Hospital have increased the rate of remuneration to their sisters from the beginning of the year. Night and theatre sisters will now receive £50 to £60; ward and casualty sisters £42 to £55. All sisters with six years' service will receive an additional £10 per annum. Staff nurses are to receive £40. The salaries of probationer nurses were raised some time ago, and now stand at £16, £18, £20, £28.

In her annual Christmas letter to her sisters and nurses, Miss Alsop, matron of the Kensington Infirmary, Lady of Grace of the Order of St. John and Jerusalem, has some earnest words on nursing reform. In expressing the need for shorter hours on duty and better conditions, she pleads for treating nurses "more like other women workers who do not have the long hours or mental strain that a nurse must endure." Miss Alsop ends on a wise note exhorting her nurses to join the College of Nursing, to encourage other nurses to become members, and to safeguard their future by joining the Royal National Pension Fund for Nurses. An admirable list of twelve reasons for joining the College of Nursing is appended to the Christmas leaflet, which has a good likeness of Miss Alsop as frontispiece.

The Edith Cavell Homes of Rest show in the recently issued report of their second year's work a tendency to develop in a rather unexpected way. There are, it appears, quite a number of people sufficiently appreciative of nursing aims to surrender their own homes to serve as centres where nursing sisters can recruit their energies. Among the offers of accommodation received by the Council we may mention those of Combe Head, opened November 11, 1917, for nine nurses; Little Wych, lent by Mrs. Nicholson, which has received eighty-two nurses in the past year; the Raven House, Adderley, near Market Drayton, for six nurses, given fully equipped and free of rates and taxes by the Hon. Mrs. Corbet; Peplow Hall, Market Drayton, where Lady Stanier receives six nurses; Southwold, lent by the Hon. Mrs. Reginald Talbot, when other homes were all full; Mythe Grange,

**ROUND THE HOSPITALS—**(*continued*).

Tewkesbury, where three guests can be received; Winton House, Richmond, where Lady Murray entertains six nurses at a time; and Henbury, near Bristol, where Mrs. Baker receives ten nurses. There are many advantages in this spontaneous hospitality offered in beautiful homes throughout the country. The Council of the Edith Cavell Homes has been very successful in eliminating possible drawbacks, and the result is of double value, to hostesses hardly less than to nurses, for it places an opportunity for rendering a specially interesting form of national service within reach. Money is still wanted to develop further the Edith Cavell Homes. They are being directed along the right lines, and in the spirit of her whose name they bear.

SOUTHPORT boasts the largest voluntary hospital for the wounded in Great Britain, and has attracted a fine body of voluntary workers to aid in their care. Dr. Bentall, in his report on the work of the St. John Hospital in that town for the year ending November 1, 1918, bears testimony to the ever increasing efficiency with which the work has been carried on, the number of cases admitted to the Grange and Woodlands Hospitals in the year having been 2,458, almost entirely of a severe type, and the deaths but eight in number. There are twenty-three professional nurses, and Dr. Bentall pays a high tribute to the efficiency of the ward sisters, to whom he attributes a large share of the success which has attended the hospitals. The V.A.D. members, of whom there are in all 400 doing nursing and general service work, have earned high praise. Eighty are on duty every day, and of these sixty-four have been regular workers since the opening three years ago, many putting in twelve hours' service a day. A movement is on foot to transfer the services of the Red Cross and V.A.D. workers to child welfare work and instruction in mothercraft. There can be no doubt that the establishment at Southport of the proposed Women's College for expert training in domestic science, commercial work, physical training, etc., would be of immense value under present conditions, and reach many whom the Devonshire House scholarship scheme is unable to help.

LINCOLN has just adopted a well-thought-out scheme for the formation of a District Nursing Association for the City of Lincoln and outlying parishes. At a meeting held at the Guildhall shortly before Christmas, the Mayor of Lincoln outlined the proposed scheme, which, he said, would cost about £1,000 a year. Part of this sum is provided already from various sources. The Bromhead Institute, which has hitherto undertaken the nursing of the sick poor in the city, will still continue to provide the nurses, but the cost of the large expansion which the staff must now undergo will be borne by the city. Much necessary work which could not be attempted in the past can be carried out under the security of increased financial support, and the city will, we trust, soon have reason to be satisfied with the new arrangements. The Board of Guardians has consented to co-operate, and has voted the sum of £50 a year.

IN the sphere of female labour the civil hospitals have much to learn from the military and auxiliary establishments. Much work of a technical and domestic kind for which men were formerly considered indispensable has been cheerfully and efficiently carried out by women, sometimes educated women, during the war. At Lambeth Infirmary an experiment was made at the beginning of 1917 in the introduction of female orderlies and porters, with most satisfactory results, in the matron's opinion. The women orderlies among other duties clean the *x*-ray and dowsing department, the operating theatre, pathological room, and dispensary; they take female patients to the wards, and move all patients from wards to theatre; they fill and empty water-beds, fetch mattresses, clean instruments, etc. The hours are 7 A.M. to 6 P.M. with the usual intervals for meals and the pay is 30*s*. a week and uniform. The portresses receive 26*s*. a week with uniform and breakfast. They have charge of the out-door premises, sweep, and clean yards, flush drains, etc., move furniture and boxes and take up all food and stores to wards. With the exception of coaling, heavy lifting, and mortuary work women are found capable of all the duties usually performed by man porters. We should be interested to have particulars from any of our readers who have tried female labour in the above direction.

THE Sister-in-Charge of the London Ambulance Column has given an interesting account of this organisation to a *Daily Telegraph* representative. The Column was set up as a branch of the B.R.C.S. for conveying the wounded from trains arriving at London termini to hospitals. Every patient who has come into London from the various battle-fronts since August 30, 1914, has been received by this organisation, which numbers 600, and consists of stretcher-bearers, drivers, nurses, and administrative staff. There are sixty ambulances and 100 motor-cars, many driven by their owners, and some 600,000 persons have been taken to their destination without hitch or delay since the beginning. They are justly proud that they have never received a complaint of any kind. The King and Queen have personally inspected the work, and expressed their approval. The work still goes on, a trainload of wounded having been received at 3.45 A.M. on New Year's Day. The average is now five trains a day, mostly repatriated prisoners.

SISTER EDITH JOHNCOCK, of the Edinburgh Medical Missionary Society, was one of the three brave nurses who remained at their posts in the city of Nazareth until carried north to Damascus by the Turks. She recounts in a letter home how the good news came through to her on October 1 of the English victory. She and her friends had been told that they were to be sent on from Damascus, and for days had been keeping in close

**ROUND THE HOSPITALS**—(*continued*).

seclusion to the house and hospital, fearing if they were seen in the street that the Turks would carry out their threat. On Michaelmas Day they heard guns and knew that fighting was going on near at hand. They had little sleep the next two days. At length, early on the morning of October 1, Sister Johncock glanced through the window of her ward and was thrilled with the sight of our men passing down the street—the first intimation they had of the British entry into the town. The wards have been overcrowded with a rush of 350 patients suffering from a bad kind of malarial fever.

WITHIN the past four years the Plymouth division of the Red Cross has posted for duty no fewer than 550 of its members as nurses, clerks, cooks, storekeepers, and general service. It has assisted to staff nine local hospitals, including the V.A. establishment at Millbay, in which it takes special pride. A great part of the success of the division is due to the fact that it was already strong when war broke out. It has successfully attracted in large measure the best kind of voluntary workers, and has been most fortunate in securing sisters able to turn this help to the best account without friction. Through the Linen League and the working parties affiliated to it some 118,000 articles for the use and comfort of the wounded have passed through the local distributing centre.

A SOCIAL meeting, interesting as being the first of a projected series of reunions, was held two days after Christmas by the members of the Aberdeen Nurses' Club and Midwives' Associations at the West End Rooms in the city. Miss Scott, the President of the Association, welcomed the guests, who numbered about 100, and the proceedings were of a varied and enjoyable nature. The success of the experiment ought to encourage the committee to arrange similar meetings, for the refreshing effects of such gatherings make them an indispensable feature in united efforts for the well-being of nurses.

MISS BERRY, matron of the Surbiton Hospital, has been through what we devoutly hope is a unique experience in finding herself the only member of her staff able to get about during the recent epidemic. With some assistance doubtless from the domestic side, she personally looked after the entire establishment, and kept the patients from experiencing any discomfort in the wreck of organised nursing. She has been presented with a silver toilet set by the governors of the hospital in recognition of her splendid courage. Perhaps an extra month's leave might also be acceptable.

THE last day of the year was fittingly chosen for a special thanksgiving service held at the Westminster Cathedral for women of the Roman Catholic faith in the national services. Many members of Queen Alexandra's Imperial Military and Naval Nursing Services attended in uniform, together with nurses of the Territorial Force Nursing Service, the British Red Cross, with V.A.D. members, private nurses, and many from civilian hospitals. Other women's national services, such as the W.R.N.S., the W.A.A.C., W.R.A.F., the Land Army, the Women Police, etc., were represented, and the scene with its bewildering variety of uniform was wonderfully picturesque in its solemn and beautiful setting. Cardinal Bourne, who was unable to be present, sent a message of special congratulation to the assembled women on their successful labours. Monsignor Howlett delivered an impressive address.

AN extraordinary account of the recent pressure in French military hospitals prior to the armistice is given by Mrs. M. Barnett McComb in the December number of the *Trained Nurse* (New York). She reports that the need for trained nurses, especially in the operating-rooms, was desperate, and she tells of an "aide" who, after being but two weeks in France, was given complete charge of a French ward containing forty-four beds "with nineteen patients coming out of ether at one time." At a base hospital in Brest she relates that the 500 beds, always occupied, were tended by one theatre nurse and two other women. It frequently took these three women two days to make the rounds and change the dressings. Long lines of wounded soldiers were to be seen moving on inch by inch towards the operating-room, some lying in the corridors thirty-six hours at a time waiting for their turn.

THE closing of California House, Lancaster Gate, for disabled Belgian soldiers marks another epoch in the passing of war conditions. All the Belgians are to be got back to their own country before the New Year is far on its way, and none have had more cause for thankfulness in the land of their adoption than those who have been aided by the American Red Cross in this institution. The funds have come chiefly from California, but Miss Heyneman, the chairman, to whom a bouquet was presented by the Belgian soldier inmates at the little farewell ceremony, said the work had been international, fully as much British as American. About 500 of the disabled have undergone treatment and received training in industrial pursuits during the last three years.

AT Christ's Hospital the authorities are very proud of the fact that not a single case of influenza occurred among the 800 boys, though it was exceedingly bad in the immediate neighbourhood. About half the boys were inoculated against it some weeks ago, and the whole school has been put through a daily course of drill, which consisted in washing out of the nose with a weak solution of permanganate of potash, with thorough nose blowing afterwards. Pending more accurate researches into the prevention of colds and influenza the public can hardly do better than follow the lead of those who successfully combat the disease, and this system of nasal washings has the merit of being so simple and inexpensive that it should prove practicable wherever infection threatens.

